# Ultrahigh-Field MRI in Human Ischemic Stroke – a 7 Tesla Study

**DOI:** 10.1371/journal.pone.0037631

**Published:** 2012-05-31

**Authors:** Vince I. Madai, Federico C. von Samson-Himmelstjerna, Miriam Bauer, Katharina L. Stengl, Matthias A. Mutke, Elena Tovar-Martinez, Jens Wuerfel, Matthias Endres, Thoralf Niendorf, Jan Sobesky

**Affiliations:** 1 Department of Neurology and Center for Stroke Research Berlin (CSB), Charité-Universitätsmedizin, Berlin, Germany; 2 Berlin Ultra-High Field Facility (B.U.F.F.), Max Delbrück Center for Molecular Medicine (MDC), Berlin, Germany; 3 Experimental and Clinical Research Center (ECRC), Charité-Universitätsmedizin and Max Delbrück Center for Molecular Medicine (MDC), Berlin, Germany; 4 NeuroCure Clinical Research Center, Charité-Universitätsmedizin, Berlin, Germany; 5 Institute of Neuroradiology, University Luebeck, Luebeck, Germany; 6 Excellence Cluster Neurocure, Charité-Universitätsmedizin, Berlin, Germany; University of Manchester, United Kingdom

## Abstract

**Introduction:**

Magnetic resonance imaging (MRI) using field strengths up to 3 Tesla (T) has proven to be a powerful tool for stroke diagnosis. Recently, ultrahigh-field (UHF) MRI at 7 T has shown relevant diagnostic benefits in imaging of neurological diseases, but its value for stroke imaging has not been investigated yet. We present the first evaluation of a clinically feasible stroke imaging protocol at 7 T. For comparison an established stroke imaging protocol was applied at 3 T.

**Methods:**

In a prospective imaging study seven patients with subacute and chronic stroke were included. Imaging at 3 T was immediately followed by 7 T imaging. Both protocols included T1-weighted 3D Magnetization-Prepared Rapid-Acquired Gradient-Echo (3D-MPRAGE), T2-weighted 2D Fluid Attenuated Inversion Recovery (2D-FLAIR), T2-weighted 2D Fluid Attenuated Inversion Recovery (2D-T2-TSE), T2* weighted 2D Fast Low Angle Shot Gradient Echo (2D-HemoFLASH) and 3D Time-of-Flight angiography (3D-TOF).

**Results:**

The diagnostic information relevant for clinical stroke imaging obtained at 3 T was equally available at 7 T. Higher spatial resolution at 7 T revealed more anatomical details precisely depicting ischemic lesions and periinfarct alterations. A clear benefit in anatomical resolution was also demonstrated for vessel imaging at 7 T. RF power deposition constraints induced scan time prolongation and reduced brain coverage for 2D-FLAIR, 2D-T2-TSE and 3D-TOF at 7 T versus 3 T.

**Conclusions:**

The potential of 7 T MRI for human stroke imaging is shown. Our pilot study encourages a further evaluation of the diagnostic benefit of stroke imaging at 7 T in a larger study.

## Introduction

Magnetic resonance imaging (MRI) using field strengths up to 3 Tesla (T) has proven to be a powerful imaging modality for the diagnosis of stroke and for the assessment of infarct morphology and stroke etiology [1]. For a few years, ultrahigh-field (UHF) MRI at 7 T has been available for imaging in humans. Recent reports demonstrated the use of UHF MRI at 7 T for brain imaging and showed relevant diagnostic benefits for brain tumors [2], cerebral malformations [3], Parkinsons's disease [4] and multiple sclerosis [5]. The sensitivity gain inherent to UHF MRI at 7 T allows for enhanced spatial resolution versus 3 T MRI and holds the promise of new diagnostic approaches for brain pathologies [6]. However, 7 T MRI is not yet available for routine clinical imaging and the potential of UHF MR for stroke imaging in clinical practice has yet to be fully realized. Preliminary experience with MRI at 7 T in stroke patients is limited to reports of time-of-flight angiography (TOF) [7] and vessel wall imaging [8]. On one hand, the sensitivity advantage inherent to MRI at 7 T offers the potential to gain new insights into stroke pathology. On the other hand, diagnostic information derived from MRI at 7 T has to be carefully validated against stroke MRI at 1.5 T or 3 T. Realizing this opportunity, our study examines the feasibility of stroke imaging at 7 T. For this reason, we propose and evaluate a clinically feasible stroke protocol for subacute and chronic stroke at 7 T. For comparison, a clinically established standard imaging protocol was used at 3 T.

## Materials and Methods

### Ethics Statement

All patients gave informed written consent prior to the study. The study was conducted according to the principles expressed in the Declaration of Helsinki and was approved by the authorized ethical review board (ERB), the governmental Berlin state ERB, and the relevant German state authority, The Federal Institute for Drugs and Medical Devices.

### Study Design

We initiated an observational prospective imaging study (7 Tesla Ultra-High Field Project, 7UP, WHO International Clinical Trials Registry No. DRKS00003193, http://apps.who.int/trialsearch/Trial.aspx?TrialID=DRKS00003193). Between August 2011 and December 2011, 298 consecutive patients either admitted with stroke to the Departments of Neurology of the Charité-Universitätsmedizin Berlin or presenting at stroke out-patient services were screened. Inclusion criteria were: 1) subacute/chronic stroke or transient ischemic attack (TIA), 2) age 18–80 years, 3) ability to give informed consent and 4) legal competence. Exclusion criteria were: 1) cardiac pacemakers or any other electronic implants, 2) metallic implants, 3) pregnancy or breast feeding period, 4) claustrophobia, 5) chronic or episodic vertigo, 6) retinal diseases and 7) dental bridges and more than two metallic dental crowns in a row. Neurological status was assessed by the National Institute of Health Stroke Scale (NIHSS) at the time of admission for index stroke and before MR imaging. Imaging was performed first at 3 T, immediately followed by imaging at 7 T. A stroke neurologist supervised the patients during imaging.

### Magnetic Resonance Imaging Hardware

MR-imaging was performed on a 3 T whole-body system (Magnetom Verio, Siemens Healthcare, Erlangen, Germany) using a 12 channel receive RF coil (Siemens Healthcare, Erlangen, Germany) tailored for head imaging. At 7 T, a whole body system (Magnetom 7T, Siemens Healthcare, Erlangen, Germany) equipped with a 90 cm bore magnet (Magnex Scientific, Oxfordshire, UK) and an Avanto gradient system (Siemens Healthcare, Erlangen, Germany) was used together with a 1/24-channel transmit/receive coil (Model NM 008-23-7S, NovaMedical, Wakefield, MA, USA) designed for head imaging.

### Magnetic Resonance Imaging Parameters

At both 3 T and 7 T the imaging protocol included the following imaging techniques:

- T1-weighted 3D Magnetization-Prepared Rapid-Acquired Gradient-Echo (3D-MPRAGE)
- T2-weighted 2D Fluid Attenuated Inversion Recovery (2D-FLAIR)
- T2-weighted 2D Turbo Spin Echo (2D-T2-TSE)
- T2* weighted 2D Fast Low Angle Shot Gradient Echo (2D-HemoFLASH)
- 3D Time-of-Flight angiography (3D-TOF).

All sequences were modified based on standard imaging protocols provided by the MR-manufacturer. T2-TSE and HemoFLASH were adapted according to [9]. Imaging parameters used at 3 T and 7 T are shown in Table 1.

**Table 1:** Synopsis of the parameters of the imaging techniques used.

7T	Sequence type	TR ms	TE ms	acceleration factor (R)	BW Hz/Px	Voxel size mm^3^	Matrix size	FA	No. of slices	t min
**MPRAGE**	3D	2750	1.81	3	350	0.7×0.7×0.7	384×384	10	17 cm coverage	05:40
**FLAIR**	2D	9000	89	1	283	1.2×0.9×3.0	256×192	130	11	03:56
**T_2_**	2D	14300	73	2	260	0.6×0.6×3.5	384×384	144	20	03:36
T_2_*	2D	600	15	3	260	0.7×0.7×3.0	320×320	25	45	02:38
**TOF**	3D	32	3.53	1	305	0.4×0.4×0.4	512×512	25	5.1 cm coverage	08:16
										Σ 24:06

3T	sequence type	TR ms	TE ms	acceleration factor (R)	BW Hz/Px	Voxel size mm^3^	Matrix size	FA	No. of slices	t min
**MPRAGE**	3D	1900	2.93	1	170	1.0×1.0×1.0	256×256	9	16 cm coverage	04:35
**FLAIR**	2D	9010	136	2	252	0.6×0.6×4.0	320×320	140	20	03:02
**T_2_**	2D	6000	96	2	220	0.7×0.7×4.0	320×320	150	25	01:20
T_2_*	2D	800	20	1	260	1.1×0.7×4.0	256×230	25	25	02:35
**TOF**	3D	24	3.60	2	186	0.6×0.6×0.6	384×364	18	8.2 cm coverage	05:54
										Σ 17:26

*TR: repetition time; TE: echo time; BW: Bandwidth; FA: flip angle; t = time.*

### Data Postprocessing and Image Analysis

Co-registration of images was performed with VINCI, Version 3.90 (Max-Planck-Institute for Neurological Research, Cologne, Germany) [10]. Visual analysis of the acquired images was performed with OsiriX, Version 4.0 (Pixmeo, Geneva, Switzerland) using a standardized qualitative evaluation including the following items (applicable sequences are listed in brackets): 1) number of lesions (FLAIR, MPRAGE, T2-TSE), 2) lesion morphology (FLAIR, MPRAGE, T2-TSE), 3) contrast between lesions and healthy tissue (FLAIR, MPRAGE, T2-TSE) and 4) assessment of the anatomical detail level (FLAIR, MPRAGE, T2-TSE, HemoFLASH). Unique information provided by specific sequences was assessed: 5) perilesional hemosiderin deposits (HemoFLASH) and 6) vessel morphology (TOF). Evaluation of these items was performed on a consensus basis by J.S. and J. W.

## Results

Seven patients (3 females, median age 42 years, interquartile range [IQR] 35–55) received imaging at 3 T and 7 T. Two patients were imaged in the subacute phase (13 and 14 days after stroke), 5 patients were imaged in the chronic phase (median years after stroke 1.6, IQR 1.3–5.2). All examinations were well tolerated by the patients, no adverse events occurred. Detailed patient data are shown in table 2.

**Table 2:** Clinical data of imaged patients.

No	Age (years)	Sex	Site of Stroke	NIHSS (admission/imaging)	Stroke to Imaging (days [d] or years [y])	No. of stroke lesions at 3 T / 7 T
1	41	m	MCA L+R	4/0	14 d	4/4
2	56	m	TIA	1/0	13 d	0/0
3	51	m	MCA L	1/0	**0.4 y**	3/3
4	28	m	MCA L	7/2	**7.2 y**	1/1
5	42	f	MCA L	3/2	**4.5 y**	1/1
6	70	f	MCA L	5/0	**1.3 y**	1/1
7	28	f	PCA L+R	0/0	**1.6 y**	2/2
Median	42			3/0	**1.6 y**	
IQR	35–55			1–5/0–2	**1.3–5.2 y**	

*m: male; f: female; MCA: medial cerebral artery; TIA: transitory ischemic attack; PCA: posterior cerebral artery, R: right; L: left; NIHSS: National Institutes of Health Stroke Scale; Stroke to Imaging: time delay between admission and MRI scans; IQR: interquartile range; in the last column only chronic patients (bold) served as basis of calculation of median and IQR.*

The mean value of the total examination time including 3D-MPRAGE, 2D-FLAIR, 2D-T2-TSE, 2D-HemoFLASH, 3D-TOF was 24:06 min at 7 T and 17:26 min at 3 T.

### 2D-FLAIR-imaging

**Figure 1:**
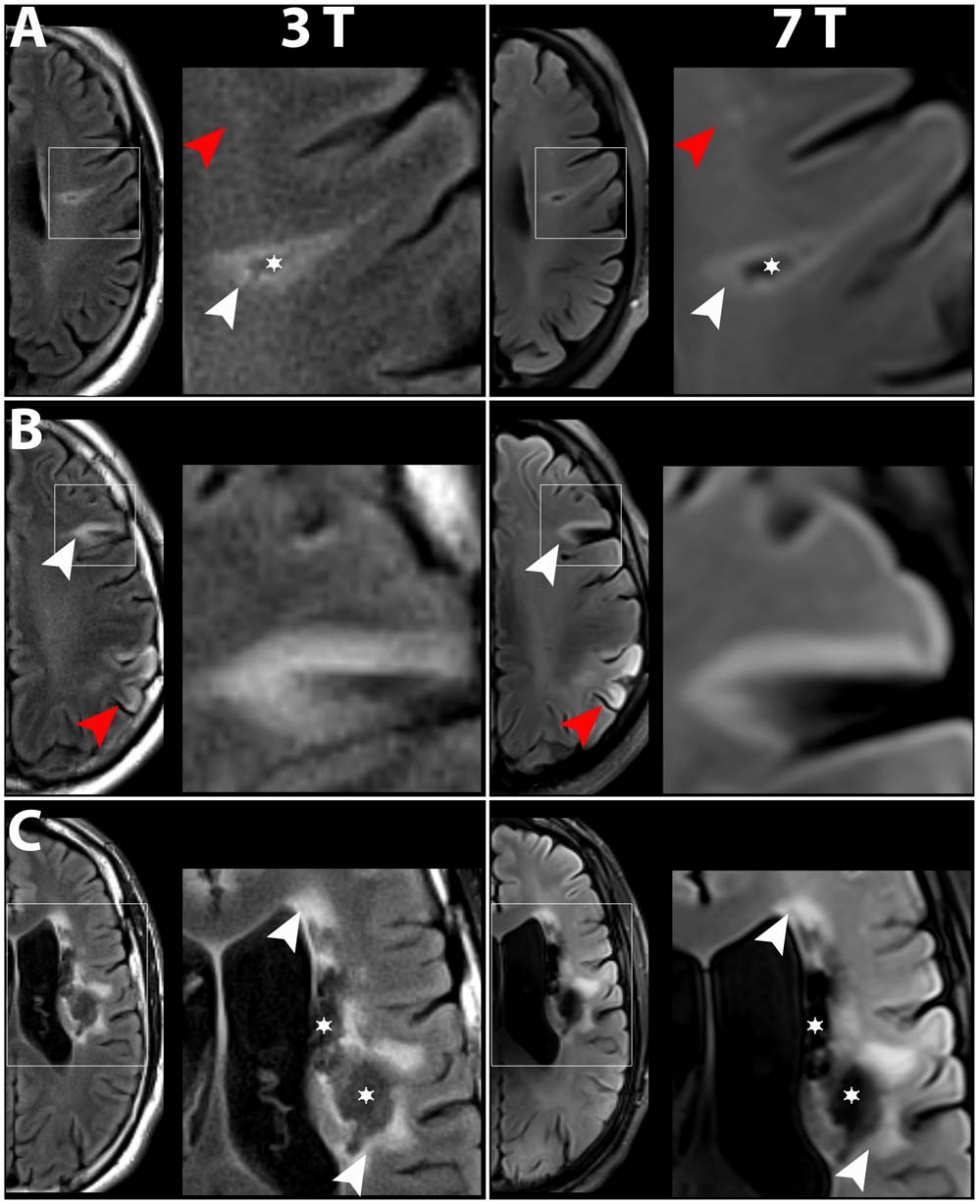
Comparison of T2-FLAIR images obtained at 3 T and 7 T. In all patients, all lesions detected at 3 T were also visible at 7 T. Boxed areas are shown at higher magnification. A) Patient No. 3, with a small chronic lesion consisting of hyperintense postischemic tissue (white arrowheads) surrounding a tissue defect area (asterisk); compare also figure 2 A. At 7 T, the intensity values of the tissue defect area were comparable to CSF, while at 3 T, the intensity values were comparable to white matter. Contrast between postischemic and healthy brain tissue was higher at 3 T. However, small white matter lesions (red arrowheads) were easier to identify at 7 T. B) Patient No. 1, with a chronic stroke lesion (white arrowheads) and a subacute lesion (red arrowheads). Both lesion types were readily identifiable at both field strengths. As in A), contrast between the lesion and healthy tissue appeared to be higher at 3 T. C) Patient No. 4, with a large chronic infarct, consisting of hyperintense lesion areas (white arrowheads) and hypointense defect areas (asterisks). Again, CSF-filled tissue defect areas were easier to identify at 7 T, while the lesion to healthy tissue contrast was higher at 3 T. Compare also fig. 2 A–C.

In all patients, the number of visible ischemic lesions was identical at both field strengths (see table 2). The depiction of the lesion morphology was superior at 7 T versus 3 T. The contrast between stroke lesions and healthy brain tissue was inferior at 7 T. In comparison, the depiction of white matter lesions (WML) at 7 T was superior owing to better contrast between WMLs and healthy tissue as demonstrated in figure 1.

### 3D-MPRAGE-imaging

**Figure 2:**
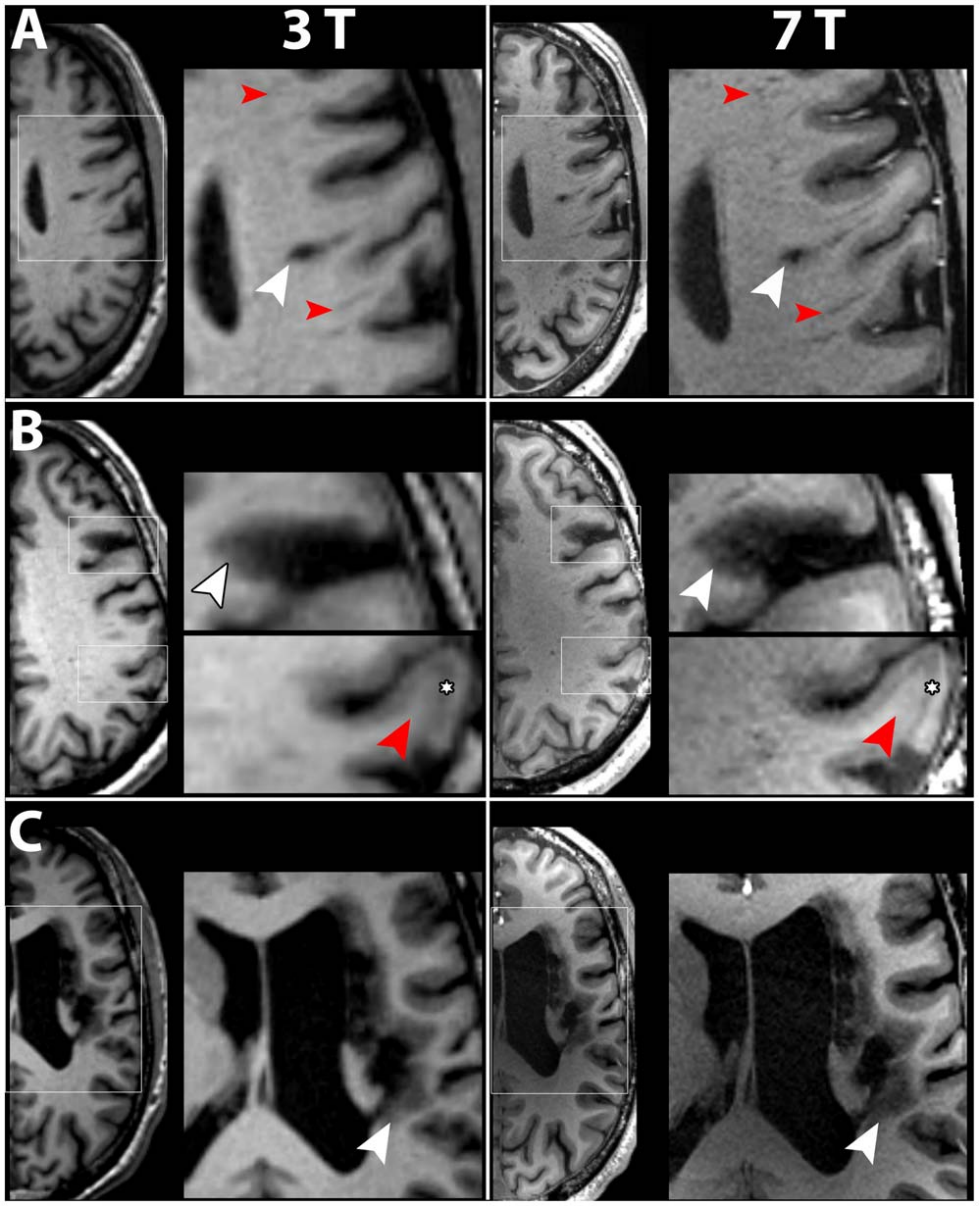
Comparison of T_1_-weighted images derived from T1-MPRAGE at 3 T and 7 T. In all patients, MPRAGE at 7 T depicted the internal structure of stroke lesion in higher detail compared with 3 T. Boxed areas are shown at higher magnification. A) Patient No. 3. The tissue defect area appeared larger and less well confined at 3 T in contrast to 7 T (white arrowheads). Virchow-Robin spaces were seen in more detail and higher frequency at 7 T (red arrowheads). B) In patient No. 1, the chronic stroke lesion (white arrowheads) presented as an hypointense area – indicating gliosis – and as a disruption of the cortical band. These characteristics of the lesion were depicted in higher detail level and contrast at 7 T. The subacute lesion (red arrowheads) showed a different internal structure of the cortical band compared with healthy cortex. Within the lesion, the cortical band was divided into a superficial hyperintense layer and a deeper hypointense layer (asterisks). Differentiation of the two layers was much easier at 7 T. C) Patient No. 4. In this large infarct, differentiation of hypointense gliosis (white arrowheads) and healthy tissue was again clearer at 7 T. Inhomogeneities between the frontal and occipital cortex and paramedian deep structures – typical for 7 T – were more pronounced in this patient compared to A) and B).

The number of visible ischemic lesions was identical at both field strengths. For all patients, MPRAGE at 7 T depicted the internal structure of stroke lesions with higher detail compared with 3 T. Contrast between lesions and healthy tissue was superior at 7 T. Small brain structures like Virchow-Robin spaces (VRS) were visualized in much higher anatomical detail and were more frequent at 7 T. Exemplary images are presented in figure 2.

### 2D-T2-TSE imaging

**Figure 3:**
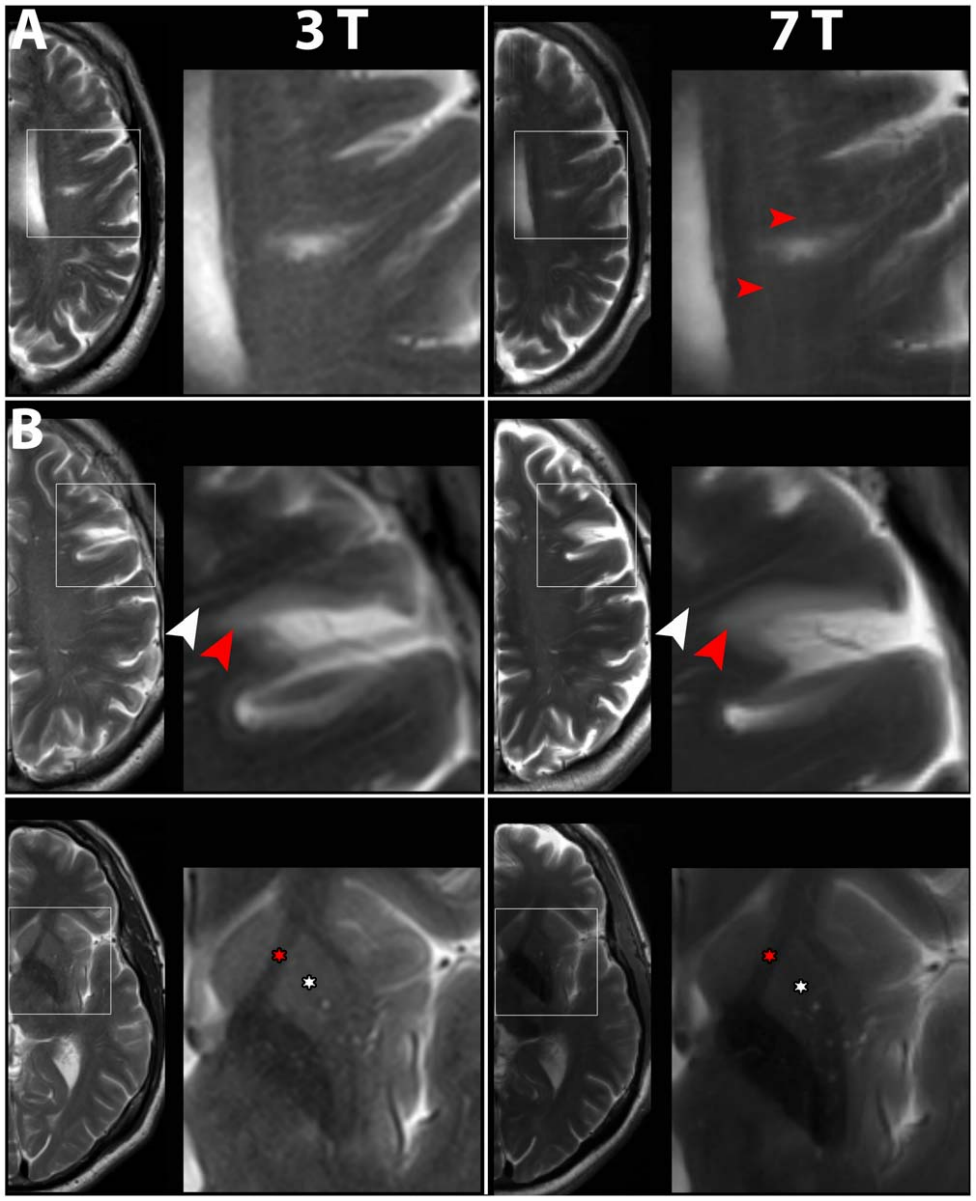
Comparison of T2-weighted imaging performed at 3 T and 7 T. Boxed areas are shown at higher magnification. A) In patient No. 3, artifacts were present (red arrowheads). Contrast and detail level of the lesion (white arrowhead) and of Virchow-Robin spaces were not higher at 7 T. B) In contrast, in patient No. 1 no artifacts were present. Virchow Robin spaces (white arrowheads) were depicted in higher detail at 7 T and the delineation of the lesion (red arrowheads) from healthy tissue was higher at 7 T. C) Same patient as in B). Also in the region of the deep nuclei T2-weighted imaging at 7 T showed better delineation, e.g. between deep nuclei (red asterisks) and fibre bundles of the internal capsule (white asterisks).

The number of visible ischemic lesions was identical at both field strengths. T2-weighted imaging at 7 T was superior to 3 T in terms of lesion morphology, visible contrast between ischemic lesions and healthy tissue/cerebrospinal fluid (CSF) and the depiction of anatomical details such as VRS. However, in 7 T T2-TSE imaging we observed artifacts in 5 out of 7 patients, which occurred exclusively for 2D T2-weighted imaging (see discussion). Exemplary images are presented in figure 3.

### 2-D-HemoFLASH imaging

**Figure 4:**
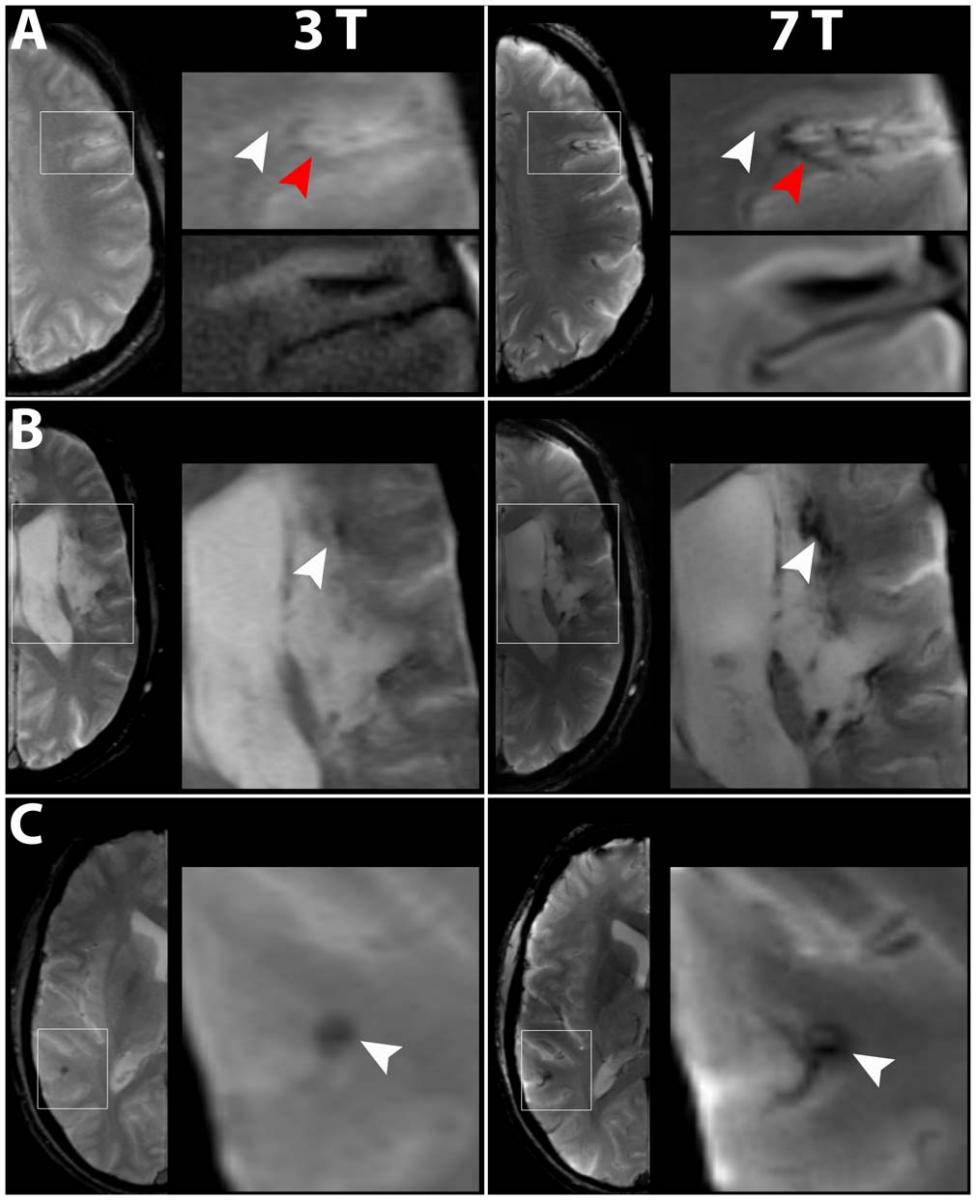
Comparison of T2*-weighted images acquired with HemoFLASH at 3 T and 7 T. In all patients, HemoFLASH provided higher anatomic detail level at 7 T. Moreover, hypointense perilesional hemosiderin deposits were much more pronounced at 7 T. Boxed areas are shown at higher magnification. A) In patient No 3, anatomical detail level and contrast of the lesion (white arrowheads) to healthy tissue were higher at 7 T imaging. T2-FLAIR weighted high magnification images are shown for comparison below. A perilesional hypointense area, indicating hemosiderin deposits, was much more pronounced at 7 T (red arrowheads). B) In patient No. 4, again both anatomical details as well as the imaging of hemosiderin (white arrowheads) were superior at 7 T. C) Incidental finding of a cavernous angioma (white arrowhead) in patient No. 5. The internal structure of the lesion, showing a nodular characteristic with a hypointense rim, and the depiction of feeding vessels were more pronounced at 7 T, facilitating the diagnosis.

In all patients, HemoFLASH at 7 T provided higher anatomical detail and allowed for a clearly better detection and delineation of perilesional hemosiderin deposits compared with HemoFLASH imaging at 3 T. In one patient (fig. 4, C), an incidental cavernous angioma was found at both field strengths. At 7 T, its internal structure and the feeding vessels were presented in much more detail compared with 3 T. Exemplary images derived from 2D-HemoFLASH imaging are depicted in figure 4.

### 3D-TOF imaging

**Figure 5:**
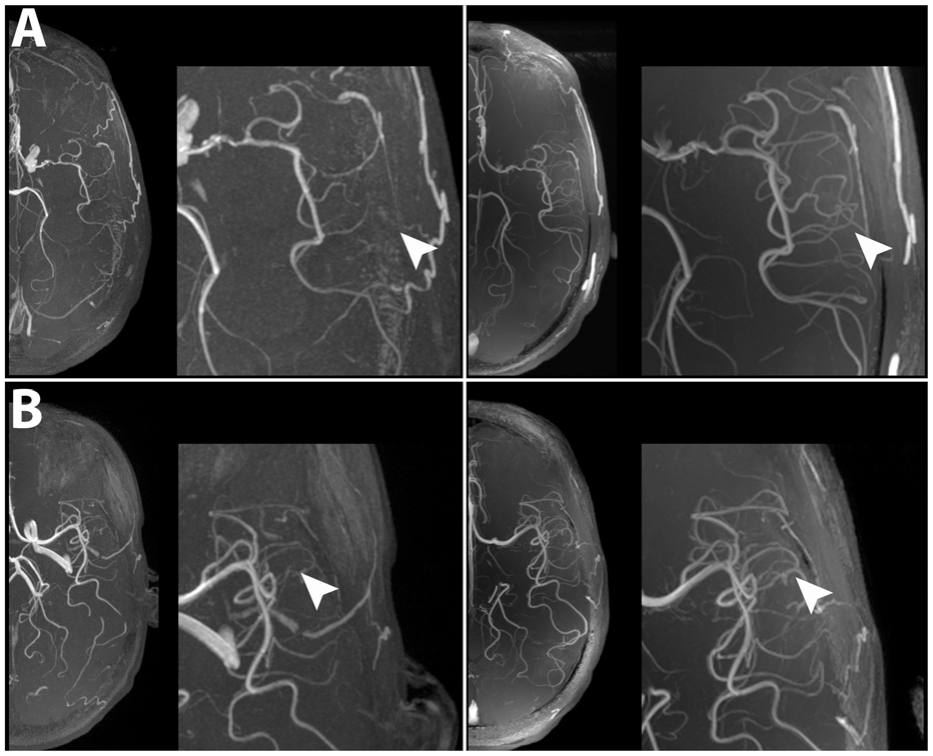
Comparison of MR-angiographies derived from 3D TOF acquisitions at 3 T and 7 T. In all patients, TOF at 7 T was able to depict the branches of the main cerebral arteries in higher anatomical detail. In patients No. 4 (A) and No. 7 (B), the left MCA territory is shown in higher magnification. In comparison with 3 T, clearly more first and second order branches were visible at 7 T in comparison with 3 T (white arrowheads).

TOF imaging at 7 T displayed more first and second order branches of the major brain arteries in all patients owing to improved lumen/background contrast in conjunction with enhanced spatial resolution. Stenoses of the intracranial arteries were not found at both field strengths. Angiographies derived from TOF acquisitions at 3 T and 7 T are shown in figure 5.

## Discussion

We report on the first head-to-head comparison of a clinically feasible stroke imaging protocol at 3 T and 7 T in subacute and chronic stroke patients. Our data suggest that the diagnostic information present at 3 T is also available at 7 T. Moreover, we found that the higher spatial resolution at 7 T reveals more anatomical details of ischemic lesions and normal brain anatomy.

Owing to improved spatial resolution and enhanced contrast mechanisms such as microscopic susceptibility-effects and T1 prolongation, MRI at 7 T promises to be beneficial for the diagnosis of neurological diseases. And indeed, previous reports on other neurological diseases using UHF-MRI were encouraging, e.g. in brain tumors [2], cerebral malformations [3] Parkinsons's disease [4] and multiple sclerosis [5]. Most of previous brain imaging studies at 7 T focused on basic research and did not regard relevant clinical issues as for example the examination time or brain coverage [8]. To establish a 7 T stroke protocol for clinical use, specific needs have to be met: First, clinical stroke imaging at 7 T has to provide the same diagnostic information, e.g. visibility of ischemic lesions, as protocols commonly used in current clinical practice at lower field strengths. Second, the total acquisition time achievable at 7 T should be comparable to established protocols at lower field strengths. Third, the imaging techniques at 7 T should be based on broadly available standard imaging protocols. From a technical point of view, RF coil design, limited availability of RF coils tailored for brain imaging, B0- and B1-field inhomogeneities, increased T1 relaxation, decreased T2 relaxation and increased RF power deposition in the patient present practical challenges at 7 T. In establishing a stroke imaging protocol for 7 T, we therefore focused on the modification of available standard protocols established at lower field strengths with the goal to balance the competing constraints of spatial resolution, RF power deposition, image quality and acquisition time.

### 2D-FLAIR imaging

Using FLAIR, all subacute and chronic lesions shown at 3 T were also readily visible at 7 T (see table 2). Given that T2-FLAIR imaging is the clinical standard for the imaging of subacute and chronic stroke lesions, our results strongly suggest that a similar contrast and therefore important diagnostic information available at 3 T is also available at 7 T. Also, we found WMLs to be better delineated from healthy tissue. However, the inversion pulse and multiple refocusing pulses intrinsic to FLAIR sequences resulted in higher RF power deposition at 7 T in comparison with 3 T. In order not to exceed RF power deposition limits, the number of slices needed to be reduced from 20 to 11 (inferior-superior brain coverage: 3.6 cm), resulting in an acquisition time that was only 54 s longer than at 3T. Without doubt, however, whole brain coverage as used at 3 T is essential for a clinical imaging protocol at 7 T. Recently introduced 3D FLAIR-techniques have the potential to overcome the limitations of the protocol used in our study [9], [11]. 3D-FLAIR will also support statistical comparison of the number of WMLs depicted at different field strengths. Recently, it has also been suggested that 7 T FLAIR might allow for the improved detection of microinfarcts [12].

On the one hand, 7 T MRI promises unique diagnostic benefits. On the other hand, it can be used for clinical imaging only, if the diagnostic information available at 3 T (or 1.5 T) can be obtained at 7 T as well. Therefore, our finding that FLAIR imaging at 7 T is equivalent to 3 T with respect to infarct detection is important and encouraging for further studies.

### 3D-MPRAGE imaging

Using MPRAGE, 7 T showed a clear improvement in the visualization of anatomical structures and lesion details at 7 T (e.g. morphology, shape and borders of the ischemic lesions and morphology and number of Virchow-Robin spaces,). To achieve a contrast comparable to 3 T, a longer TR was chosen at 7 T due to T1 prolongation. Using parallel imaging (acceleration factor = 2) our MPRAGE acquisition time was only slightly increased compared with 3 T without sacrificing the spatial resolution advantage over the 3 T approach. Moreover, our protocol facilitated RF power deposition levels, which did not exceed the limits of the normal operating mode. Consequently, whole brain coverage MPRAGE at 7 T was feasible for our protocol. MPRAGE at 7 T as used in our protocol therefore offers an unprecedented depiction of anatomical details of stroke lesions. As a result, the correlation of stroke location and neurological symptoms especially in brain regions with a high density of nuclei with different functions, e.g. the thalamus, will be greatly facilitated. Furthermore, the number and enlargement of VRS may be correlated with aging, dementia and signs of small vessel disease, a risk factor of stroke [13]–[15]. MPRAGE at 7 T seems to be better suited to assess number and size of VRS in stroke patients than imaging at lower field strengths, thus potentially facilitating research in this area.

### T2-weighted imaging

For T2-weighted imaging, stroke lesions and small brain structures like VRS could be depicted in higher detail at 7 T in comparison with 3 T. In contrast to the other techniques used we observed image artifacts for T2-weighted imaging at 7 T, which can be attributed to bulk head motion. Given that other sequences were not affected, adaptation of motion artifact reducing techniques [16], [17] will help to further improve the value of T2-TSE-imaging at 7 T. T2-weighted TSE at 7 T exhibits high RF power deposition owing to the use of a train of refocusing RF pulses. We were able to acquire 20 slices per patient (superior-inferior [S-I] coverage: 7 cm), which can be considered sufficient, if the site of the lesion is known. However, more slices or even whole brain coverage would be desirable in the future and acquisition time at 7 T was almost 3 times longer than at 3 T (3:36 min vs 1:20). Here, the use of RF refocusing pulses lower than 180 degrees or the application of specific absorption rate saving techniques, which modulate the amplitude of the refocusing RF pulses [18], [19], can be helpful to offset RF power deposition constraints.

### 2D-HemoFLASH

HemoFLASH is a 2D FLASH sequence with relatively long TE sensitive for blood and blood products like hemosiderin (9). In stroke lesions, the detection of hemosiderin deposits and microbleeds is of major importance [20], [21]. HemoFLASH at 7 T was clearly superior to HemoFLASH at 3 T in terms of spatial resolution, anatomical detail and in depicting perilesional hemosiderin deposits. Moreover, with parallel imaging (acceleration factor = 3) the acquisition time at 7 T was comparable to 3 T, while whole brain coverage was achieved with more slices than at 3 T (45 vs. 25). Considering the superior depiction of an incidentally found cavernous angioma at 7 T, our results suggest potential diagnostic benefits using HemoFLASH imaging at 7 T in the future.

### 3D-TOF

TOF at 7 T was able to show more first and second order branches of the major vessels in our study. These results in stroke patients are in accordance with previous reports on TOF-imaging at 7 T [7], [22]. TOF is challenging at 7 T owing to high RF power deposition that leads to long acquisition times and to an incomplete brain coverage (S-I coverage is approximately 5 cm at 7 T vs. 8 cm at 3 T, when using the imaging paramters shown in table 2). This renders 7 T currently suitable for a detailed visualization of selected brain regions but not for a screening method of the whole basal intracranial vessel system. Moreover, since no patient in our sample showed vessels stenoses, the potential of TOF at 7 T for the imaging of intracranial macroangiopathy remains to be evaluated. Given the superiority in terms of spatial resolution, however, a clear diagnostic benefit in the imaging of diseases with affection of macro-vessels, eg. atherosclerosis or cerebral vasculitis, can be expected in the future.

In summary, 7 T offered substantial benefits over 3 T imaging in stroke with respect to improved spatial resolution and depiction of the infarct and vessel morphology as well as stroke related pathological findings such as hemosiderin deposits. These findings substantiate a preliminary report on stroke imaging at 8 T, where diagnostic advantages over imaging at 1.5 T were described [23]. The introduction of promising new technologies such as 7 T requires the proof of a diagnostic benefit over routinely used methods to justify the translation into clinical use. Our initial results show benefits in several aspects of 7 T MRI for the imaging of ischemic lesions and intracranial vessels. Thus, our data favor a further exploration of UHF MRI at 7 T to investigate the specific benefit for stroke imaging. However, we also identified technological and practical challenges such as limited brain coverage owing to SAR constraints, longer acquisition times and susceptibility to motion (T2-imaging), which need to be solved before clinical use. These technical improvements are part of our ongoing study.

Our preliminary study has several limitations. First, we present a limited number of patients that precludes a concluding judgment of the value of 7 T MRI. Given that almost 300 patients were screened for our study, the number of patients imaged reflects the cautious exclusion criteria, which have to be applied to UHF MRI at 7 T according to our ERB approval. However, our study reports on a unique comparison of 3 T and 7 T imaging in stroke and serves as a pilot study to inspire further research explorations into the challenges and benefits of stroke imaging at ultrahigh magnetic fields.

Second, owing to the strict exclusion criteria the median age of the patients imaged in our study (42 a) was lower and thus not representative for the general stroke population. Along with the increasing use of 7 T MRI and the advancing clinical experience, the use of stroke imaging at 7 T in a broader patient population is to be expected.

Third, our comparison is a visual and therefore qualitative analysis. Our study is ongoing and with increasing patient numbers, a quantitative approach will be targeted.

Fourth, the diagnostic value of MRI mainly depends on the imaging parameters as well as the scanning time. The 7 T protocol – while clinically acceptable – yielded an acquisition time which exceeded that of the 3 T protocol (24 min vs 17 min). Further emphasis on shortening the acquisition times, while maintaining superior spatial resolution without losing diagnostic information, is still necessary and part of our ongoing investigations.

Fifth, only subacute and chronic stroke patients were included in this study. In the future, the value of 7 T MRI should be evaluated in acute stroke patients. Our study paves the way for the clinical use of 7 T MRI in stroke. However, diffusion weighted imaging (DWI), the clinical standard of acute stroke MR imaging, does not offer sufficient image quality at 7 T yet (8) and will need further development.

Sixth, most of the patients imaged had mild strokes with small lesions. With broader availability of 7 T imaging, better accessibility of 7 T MRI for patients with more severe disabilities can be expected.

In conclusion, stroke lesions visible at 3 T can all be detected at 7 T. 7 T imaging of stroke provides improved spatial resolution which helps to reveal more anatomical detail and pathophysiological characteristics of ischemic lesions. Our findings encourage a further exploration of the diagnostic benefit 7 T MRI may offer for the imaging of stroke. RF power, acquisition time and limited brain coverage constraints present challenges in some of the sequences for stroke imaging at 7 T, but should inspire basic researchers and clinical scientists to throw further weight behind the solution of the remaining technical problems.

## References

[1] Merino JG, Warach S (2010). Imaging of acute stroke.. Nat Rev Neurol.

[2] Lupo JM, Li Y, Hess CP, Nelson SJ (2011). Advances in ultra-high field MRI for the clinical management of patients with brain tumors.. Current Opinion in Neurology.

[3] Dammann P, Barth M, Zhu Y, Maderwald S, Schlamann M (2010). Susceptibility weighted magnetic resonance imaging of cerebral cavernous malformations: prospects, drawbacks, and first experience at ultra–high field strength (7-Tesla) magnetic resonance imaging.. Neurosurgical FOCUS.

[4] Kwon D, Kim J, Oh S, Jeong H, Park S (2012). Seven-tesla magnetic resonance images of the substantia nigra in Parkinson disease. Annals of Neurology, Annals of Neurology 71, 71: 267, 267–277, 277.. doi:10.1002/.

[5] Tallantyre EC, Dixon JE, Donaldson I, Owens T, Morgan PS (2011). Ultra-high-field imaging distinguishes MS lesions from asymptomatic white matter lesions.. Neurology.

[6] Qian Y, Zhao T, Zheng H, Weimer J, Boada FE (2011). High-resolution sodium imaging of human brain at 7 T. Magnetic Resonance in Medicine..

[7] Kang C, Park C, Park C, Lee Y, Cho Z (2010). Research: Lenticulostriate arteries in chronic stroke patients visualised by 7 T magnetic resonance angiography.. International Journal of Stroke.

[8] van der Kolk AG, Zwanenburg JJM, Brundel M, Biessels G-J, Visser F (2011). Intracranial Vessel Wall Imaging at 7.0-T MRI.. Stroke.

[9] Wiggins GC, Sodickson DK (2011). Towards Clinical 7T MRI.. Magnetom Flash 46.

[10] Cízek J, Herholz K, Vollmar S, Schrader R, Klein J (2004). Fast and robust registration of PET and MR images of human brain.. NeuroImage.

[11] de Graaf WL, Zwanenburg JJM, Visser F, Wattjes MP, Pouwels PJW (2012). Lesion detection at seven Tesla in multiple sclerosis using magnetisation prepared 3D-FLAIR and 3D-DIR.. Eur Radiol.

[12] Brundel M, de Bresser J, van Dillen JJ, Kappelle LJ, Biessels GJ (2012). http://dx.doi.org/10.1038/jcbfm.2011.200.

[13] Zhu Y-C, Dufouil C, Mazoyer B, Soumaré A, Ricolfi F (2011). Frequency and Location of Dilated Virchow-Robin Spaces in Elderly People: A Population-Based 3D MR Imaging Study.. American Journal of Neuroradiology.

[14] Zhu Y-C, Tzourio C, Soumaré A, Mazoyer B, Dufouil C (2010). Severity of Dilated Virchow-Robin Spaces Is Associated With Age, Blood Pressure, and MRI Markers of Small Vessel Disease.. Stroke.

[15] Chen W, Song X, Zhang Y, for the Alzheimer's Disease Neuroimaging Initiative (2011). Assessment of the Virchow-Robin Spaces in Alzheimer Disease, Mild Cognitive Impairment, and Normal Aging, Using High-Field MR Imaging.. American Journal of Neuroradiology.

[16] Pipe JG (1999). Motion correction with PROPELLER MRI: Application to head motion and free-breathing cardiac imaging. Magnetic Resonance in Medicine 42: 963–969.. doi:10.1002/(SICI)1522-2594(199911)42:5<963:: AID-MRM17>3.0.CO;2-.

[17] Nyberg E, Sandhu G s, Jesberger J, Blackham K a, Hsu D p (2012). Comparison of Brain MR Images at 1.5T Using BLADE and Rectilinear Techniques for Patients Who Move during Data Acquisition.. American Journal of Neuroradiology.

[18] Hennig J, Scheffler K (2001). Hyperechoes.. Magnetic Resonance in Medicine.

[19] Busse RF (2004). Reduced RF power without blurring: Correcting for modulation of refocusing flip angle in FSE sequences.. Magnetic Resonance in Medicine.

[20] Weber R, Wegener S, Ramos-Cabrer P, Wiedermann D, Hoehn M (2005). MRI detection of macrophage activity after experimental stroke in rats: New indicators for late appearance of vascular degradation?. Magnetic Resonance in Medicine.

[21] Bokura H, Saika R, Yamaguchi T, Nagai A, Oguro H (2011). Microbleeds Are Associated With Subsequent Hemorrhagic and Ischemic Stroke in Healthy Elderly Individuals.. Stroke.

[22] Heverhagen JT, Bourekas E, Sammet S, Knopp MV, Schmalbrock P (2008). Time-of-flight magnetic resonance angiography at 7 Tesla.. Invest Radiol.

[23] Novak V, Abduljalil AM, Novak P, Robitaille PM (2005). High-resolution ultrahigh-field MRI of stroke.. Magnetic Resonance Imaging.

